# Construction of a prognostic prediction model for renal clear cell carcinoma combining clinical traits

**DOI:** 10.1038/s41598-023-30020-4

**Published:** 2023-02-27

**Authors:** Yujie Weng, Pengfei Ning

**Affiliations:** College of Computer and Information, Inner Mongolia Medical University, Hohhot, 010110 Inner Mongolia Autonomous Region China

**Keywords:** Cancer, Computational biology and bioinformatics

## Abstract

Kidney renal clear cell carcinoma (KIRC) is one of the common malignant tumors of the urinary system. Patients with different risk levels are other in terms of disease progression patterns and disease regression. The poorer prognosis for high-risk patients compared to low-risk patients. Therefore, it is essential to accurately high-risk screen patients and gives accurate and timely treatment. Differential gene analysis, weighted correlation network analysis, Protein–protein interaction network, and univariate Cox analysis were performed sequentially on the train set. Next, the KIRC prognostic model was constructed using the least absolute shrinkage and selection operator (LASSO), and the Cancer Genome Atlas (TCGA) test set and the Gene Expression Omnibus dataset verified the model’s validity. Finally, the constructed models were analyzed; including gene set enrichment analysis (GSEA) and immune analysis. The differences in pathways and immune functions between the high-risk and low-risk groups were observed to provide a reference for clinical treatment and diagnosis. A four-step key gene screen resulted in 17 key factors associated with disease prognosis, including 14 genes and 3 clinical features. The LASSO regression algorithm selected the seven most critical key factors to construct the model: age, grade, stage, GDF3, CASR, CLDN10, and COL9A2. In the training set, the accuracy of the model in predicting 1-, 2- and 3-year survival rates was 0.883, 0.819, and 0.830, respectively. The accuracy of the TCGA dataset was 0.831, 0.801, and 0.791, and the accuracy of the GSE29609 dataset was 0.812, 0.809, and 0.851 in the test set. Model scoring divided the sample into a high-risk group and a low-risk group. There were significant differences in disease progression and risk scores between the two groups. GSEA analysis revealed that the enriched pathways in the high-risk group mainly included proteasome and primary immunodeficiency. Immunological analysis showed that CD8 (+) T cells, M1 macrophages, PDCD1, and CTLA4 were upregulated in the high-risk group. In contrast, antigen-presenting cell stimulation and T-cell co-suppression were more active in the high-risk group. This study added clinical characteristics to constructing the KIRC prognostic model to improve prediction accuracy. It provides help to assess the risk of patients more accurately. The differences in pathways and immunity between high and low-risk groups were also analyzed to provide ideas for treating KIRC patients.

## Introduction

Renal cell carcinoma is one of the common malignant tumors of the urinary tract, which originates from the urinary tubular epithelial system of the renal parenchyma, of which KIRC is one of the most common subtypes, accounting for about 70–80%^1,2^. Currently, the incidence of KIRC is increasing yearly, and it is one of the top 10 cancers that cause death in patients^3,4^. Patients with different risk levels differ in the disease’s cause, progression, treatment, and prognosis. Targeted treatment can be administered if the patient’s risk level can be accurately distinguished. Therefore, it is necessary to construct a prognostic risk model based on the patient’s gene expression level and clinical characteristics.

Lasso regression is a compression estimation method with the idea of reducing the set of variables^5^. It can compress the coefficients of variables and make some regression coefficients zero by constructing a penalty function, thus achieving variable selection^6^. Biological data are high-dimensional and therefore require more samples. Otherwise, the model tends to be over-fitted. In contrast, the lasso can filter the variables and reduce the complexity of the model, and does not require high data samples, which is suitable for model construction of biological data^7^.

Many investigators have used Lasso to construct prognostic models for KIRC. Using Lasso Cox regression analysis, Liu et al. made a metabolism-related prognostic model based on ten genes. The accuracy rate is 0.708^8^. Meng et al. constructed a prediction model based on five alternative splicing events associated with overall survival screened by Lasso regression with an area under the curve of 0.788^9^. Huang et al.^10^ identified three significant prognostic factors, ALDOB, EFORD1, and ESRRG, in KIRC patients by multivariate Cox regression analysis with an area under the AUC curve of 0.717. Hu et al.^11^ constructed a model based on seven methylated expressed genes with an accuracy of 0.798. Wu et al.^12^ developed a prognostic risk model using seven TRIM family genes with an accuracy of 0.685. Researchers have constructed models from different perspectives, but the accuracy of the models is not very high, which leaves much uncertainty in predicting patients’ risk.

In this study, the prognostic model was constructed using the KIRC key genes and clinical features to improve the accuracy of the prognostic model further. The accuracy of 1-year survival prediction was 0.883 in the training set and 0.831 and 0.812 in the test set, respectively. The results show that the model has high accuracy in different datasets and can be used as a valid model to predict the prognosis of KIRC patients in clinical. Accurate and effective prediction of a patient’s risk level allows targeted treatment. Also, being able to provide better assistance to patients can prevent over-treatment.

## Materials and methods

### Data sets and data processing

The datasets were obtained from the TCGA (https://portal.gdc.cancer.gov/) database and the GEO (https://www.ncbi.nlm.nih.gov/geo/) database, respectively^13,14^. The TCGA dataset includes both transcriptomic and clinical data, with 539 KIRC tumor samples and 72 normal samples in the transcriptomic data, for a total sample size of 611. Clinical data samples were downloaded in 537 cases. The sample name, survival time, survival status, age, sex, T, M, N, grader, and stage information were extracted from the downloaded files in Perl language. The transcriptomic and clinical data were combined by sample name; the combined dataset was 519 cases.

The dataset downloaded from the GEO database is GSE29609. The gene expression and clinical dataset were extracted using the GEOquery package in R. The clinical data set was processed to collate survival time and status, T, M, N, and grade information. After combining the GEO transcriptomic data and clinical data by sample name, the sample size was 39 cases.

The above sample sets were grouped, and 300 samples from the TCGA database were randomly selected as the train set. The remaining 219 samples and 39 samples from GSE29609 were used as the test set for validation, which was used to evaluate the accuracy of the prognostic model.

### Differential gene screening

Differential gene expression analysis between the normal and cancer groups was done using the limma package of R software^15^.The expression data are first preprocessed, and if multiple rows exist for a gene, then the average value is taken as its expression value. If the gene expression values are all 0, then delete them. The partial data were corrected using the log2 transformation in the limma package, and the voom method was used for normalization to adjust for errors due to the gene microarray technique. The data were divided into normal and tumor samples, and the rank sum test was performed using the Wilcox function to analyze whether there was a difference in the means of the two data groups. The filtering conditions for differential genes were |logFC| (log fold change) > 2 and FDR (false discovery rate) < 0.001^16^.

### WGCNA analysis screening key module

WGCNA analysis was done on the obtained differential genes, and the set of genes with the most significant correlation with clinical traits was obtained by combining the grouping information of the samples^17^. The gene expression and trait matrix are first prepared, and the data are rechecked to remove missing and outliers. Next, a soft threshold of 6 was chosen based on the near-scale-free topology criterion. BlockwiseModules function was used to construct the network to obtain the number of modules divided and the number of genes inside each module. Finally, the modules are correlated with the phenotypic data to obtain modules significantly correlated with the phenotypic data.

### PPI network visualization and screening of key genes

The genes in the maximum relevance module in WGCNA were exported and imported into the string (https://cn.string-db.org/) database to obtain the protein interworking network. The network was exported and imported into cytoscape software for visualization^18^. In cytoscape, the molecular complex detection (MCODE) method obtains key sub-networks and genes.

### Univariate Cox regression analysis for key prognostic factors

The Cox regression model (proportional hazards model) uses survival outcome and survival time as dependent variables and can analyze the effect of multiple factors on survival at the same time and obtain the result of whether each character is related to survival (*p* < 0.05). This study used univariate Cox analysis to screen key genes and clinical characteristics (survival status, survival time, age, sex, grade and stage) to obtain key factors associated with prognosis^19^. Meanwhile, the prognostic key genes were validated on the GEPIA website (http://gepia.cancer-pku.cn/)^20^.

### Construction of the model

The model is constructed first by calculating the weights from the effect of each key factor on the outcome. Next, all key elements are combined to derive a risk score for each patient. Finally, the patient’s risk level is assessed based on the risk score.

LASSO regression allows for a linear model between key factors and prognostic outcomes, with variable screening and complexity adjustment^21^. Variable screening refers to selectively putting variables into the model to get better results, and complexity adjustment refers to controlling the complexity of the model through parameters to avoid over-fitting^22^. LASSO regression was implemented using the glmnet package in R to construct prognostic key factors and prognostic outcomes model. The sample was divided into high-risk and low-risk groups based on risk scores. To verify the model’s validity, compare the difference in survival time and survival status between high and low-risk groups. Create Cox curves to see the accuracy of the model. Considering that gene expression, treatment, and outcome variables at different stages and grades, the models were constructed separately at each grade and stage.

### Test set to verify model validity

Model validation is a way to assess whether the model is valid. The obtained model calculates a risk score for each patient in the test dataset. The risk score is used to get the patient’s risk rating. If it agrees with the actual risk level of the patient, it indicates that the model is valid.

Model validation in test sets. There are 2 test sets, the TCGA test set, which includes 219 samples, and the GSE29609 test set, which consists of 39 samples. According to the formula for risk scoring, risk scores were calculated in the sample of the test set, and the sample was divided into a high-risk group and a low-risk group according to the median value to see if there were significant differences between the two groups in terms of survival time and survival status.

To further verify the model’s validity, the training and testing sets of TCGA were divided into different groups according to age, grade, and stage. See if the model is valid in various groupings. Also, the GSE29609 dataset was grouped by tumor necrosis, sarcomatoid component, renal vein involvement, renal vein involvement, microvascular invasion, and death to see if there were differences in risk scores among the different groupings.

### GSEA enrichment analysis for high and low risk groups

The samples were divided into high and low-risk groups, with the high-risk group as a comparison group and the low-risk group as a control group for GSEA enrichment analysis^23^. The different pathways enriched in the high and low-risk groups can suggest the cause of the disease and provide some new ideas for treatment.

### Immunological analysis of high and low risk groups

In the high- and low-risk groups, immune correlation analysis was used to look at differences in immune cells, immune function, and immune checkpoints in the high- and low-risk groups. The correlation analysis of immune cells requires using the immune cell infiltration results file for TCGA samples downloaded from the timer (http://timer.cistrome.org) website^24^.The file includes the results calculated by six methods: CIBERSORT, CIBERSORT-ABS, QUANTISEQ, MCPCOUNTER, XCELL, and EPIC. Immune function analysis is implemented using the gsva package in the R language, which converts gene expression data into scoring files for immune-related functions^25^.

Immune checkpoint inhibitor therapy has emerged as an effective tool for the immunotherapy of tumors^26^. The samples were divided into high-risk and low-risk groups to see if the expression of immune checkpoint-related genes differed, thus providing some new ideas for the immunotherapy of KIRC.

## Results

### Study design

**Figure 1:**
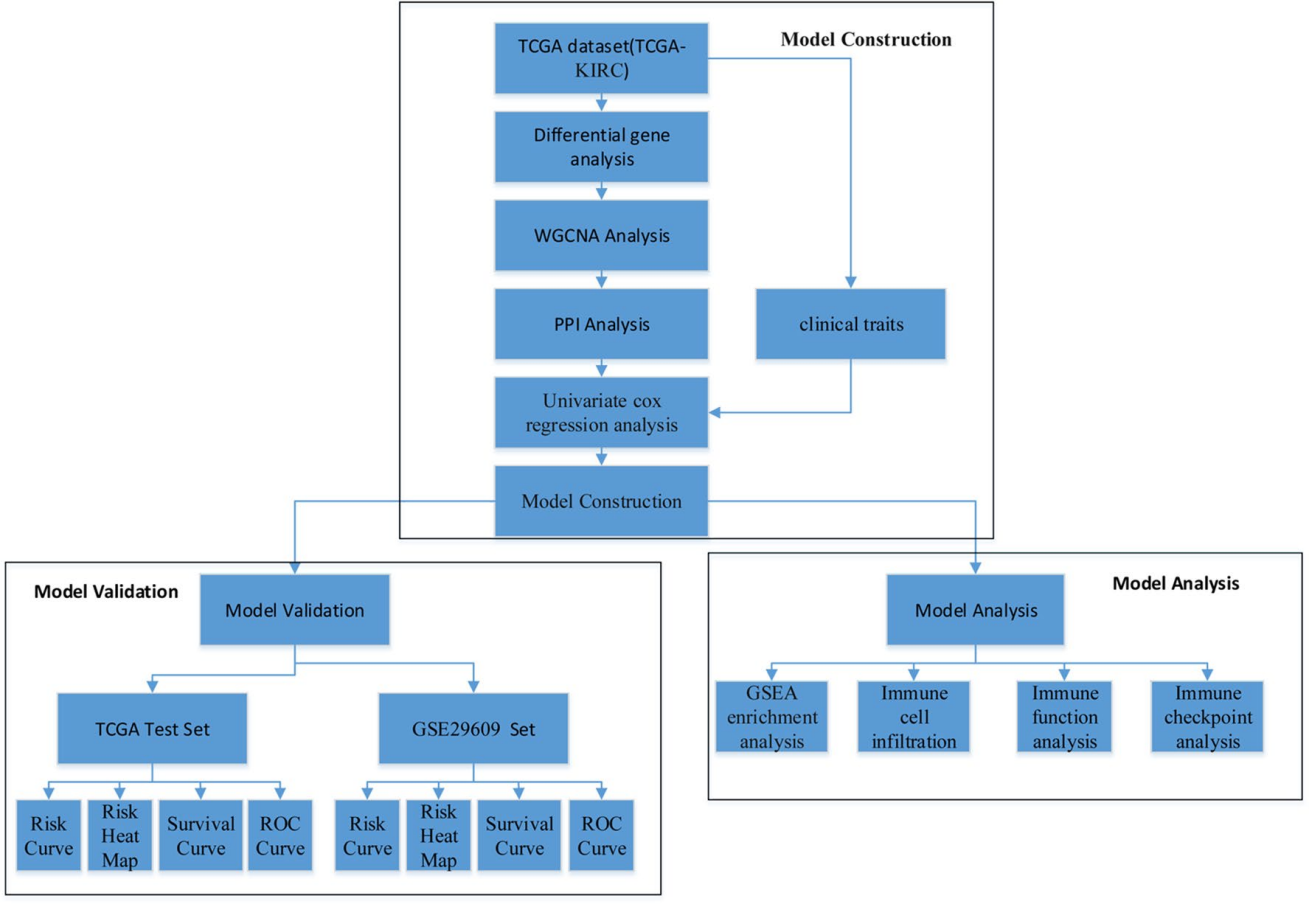
Design flow chart. The design includes three parts, model construction, model validation, and model analysis.

The design flow chart of this study is shown in Fig. 1. We downloaded transcriptomic and clinical data from TCGA-KIRC, and 519 samples were matched and filtered. The samples were divided into 300 training sets and 219 test sets, while GSE29609 was downloaded as an external test set. The overall design includes three parts: model construction, model validation, and model analysis.

First, a set of key genes were obtained in the model construction part by differential gene analysis, WGCNA analysis, and PPI network. Secondly, univariate Cox regression analysis of key genes combined with clinical characteristics was performed to screen out key genes and clinical characteristics associated with prognosis. Finally, a multifactorial prognostic model was constructed using LASSO regression.

In the model validation part, the test set of TCGA and GSE29609 datasets were used for validation, including the risk curve, risk heat map, survival curve, and ROC curve.

In the model analysis section, the samples were divided into high and low-risk groups using the prognostic model we obtained, and the differences in pathways, immune cell infiltration, immune function, and immune checkpoints between the high and low-risk groups were analyzed to provide some ideas for clinical diagnosis and treatment.

### Differential gene screening

**Figure 2:**
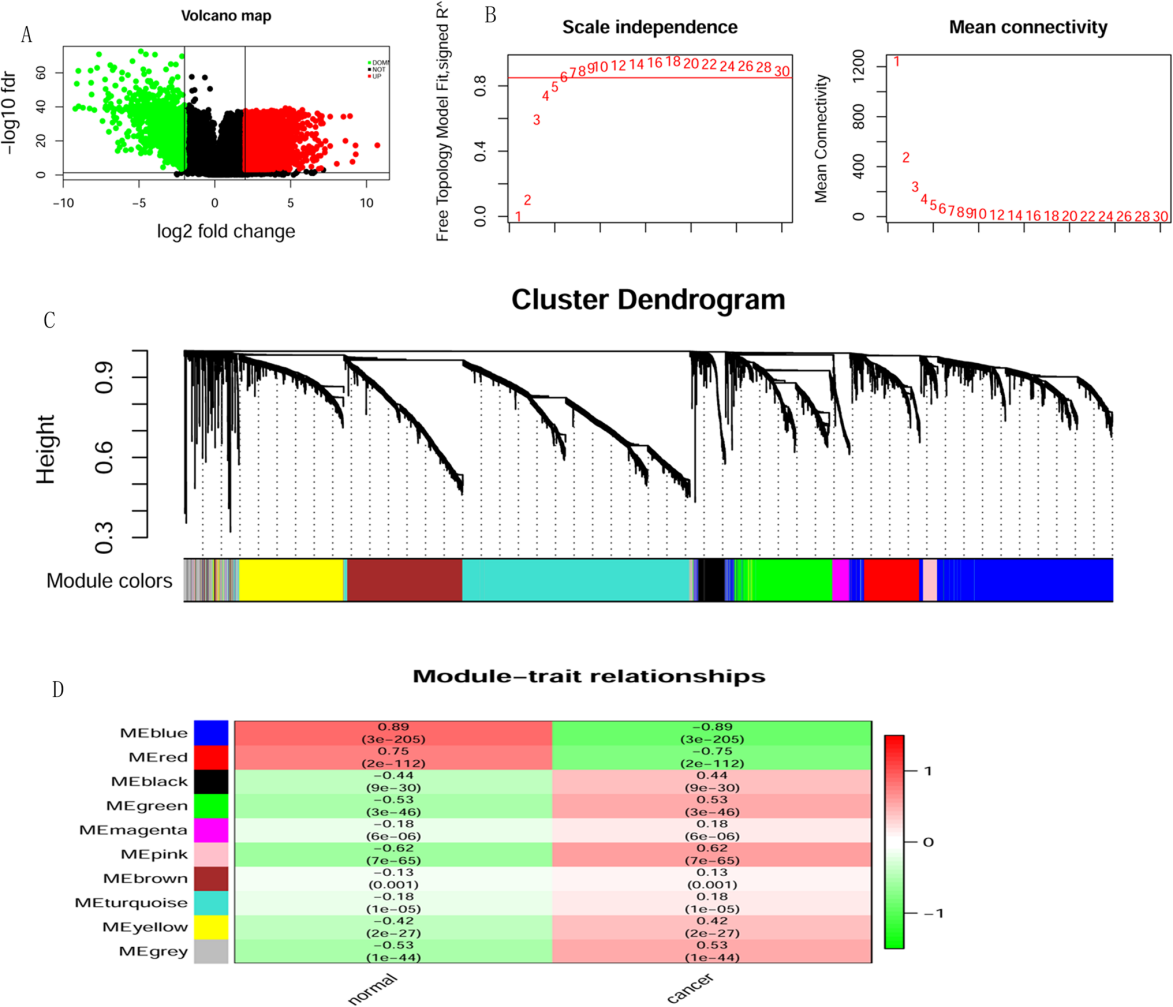
Results of DEGs and WGCNA analysis. (**A**) Volcano. The red part indicates the genes highly expressed in KIRC, and the green part indicates the genes with low expression. (**B**) The soft threshold for filtering WGCNA using the pickSoftThreshold method is 6. (**C**) The clustering algorithm divides the genes into ten different modules, and different colors in the clustering number in the figure represent different modules. (D) Module trait relationships. Green indicates a negative correlation; red indicates a positive correlation. The graph’s blue module (MEblue) has the largest negative correlation with the trait, and the pink module (MEpink) has the largest positive correlation.

The limma algorithm of the R package was used to do differential gene analysis on TCGA-KIRC data using |logFC| > 2 and FDR < 0.001 as the screening condition for differential genes. A total of 5695 differentially expressed genes were obtained (Fig. 2A). Among them, 1341 were down-regulated, and 4354 were up-regulated. Considering that DEGs are the first step of genetic screening and the scope will be gradually narrowed down, the screening conditions are relatively lenient.

### WGCNA analysis screening key module

WGCNA analysis was done on 5695 differential genes to obtain the module with the most significant correlation with the grouping information. A co-expression network was constructed by soft thresholding6 (Fig. 2B), and ten modules were identified, represented by different colors (Fig. 2C). The correlation between gene modules and grouping information was calculated. The most extensive negatively correlated module (blue module) and the largest positively correlated module (pink module) were identified separately, as shown in Fig. 2D. The blue module had 1242 genes with a maximum correlation coefficient of 0.89. The pink module had 105 genes with a correlation coefficient of 0.62.

### PPI network visualization and screening of key genes

**Figure 3:**
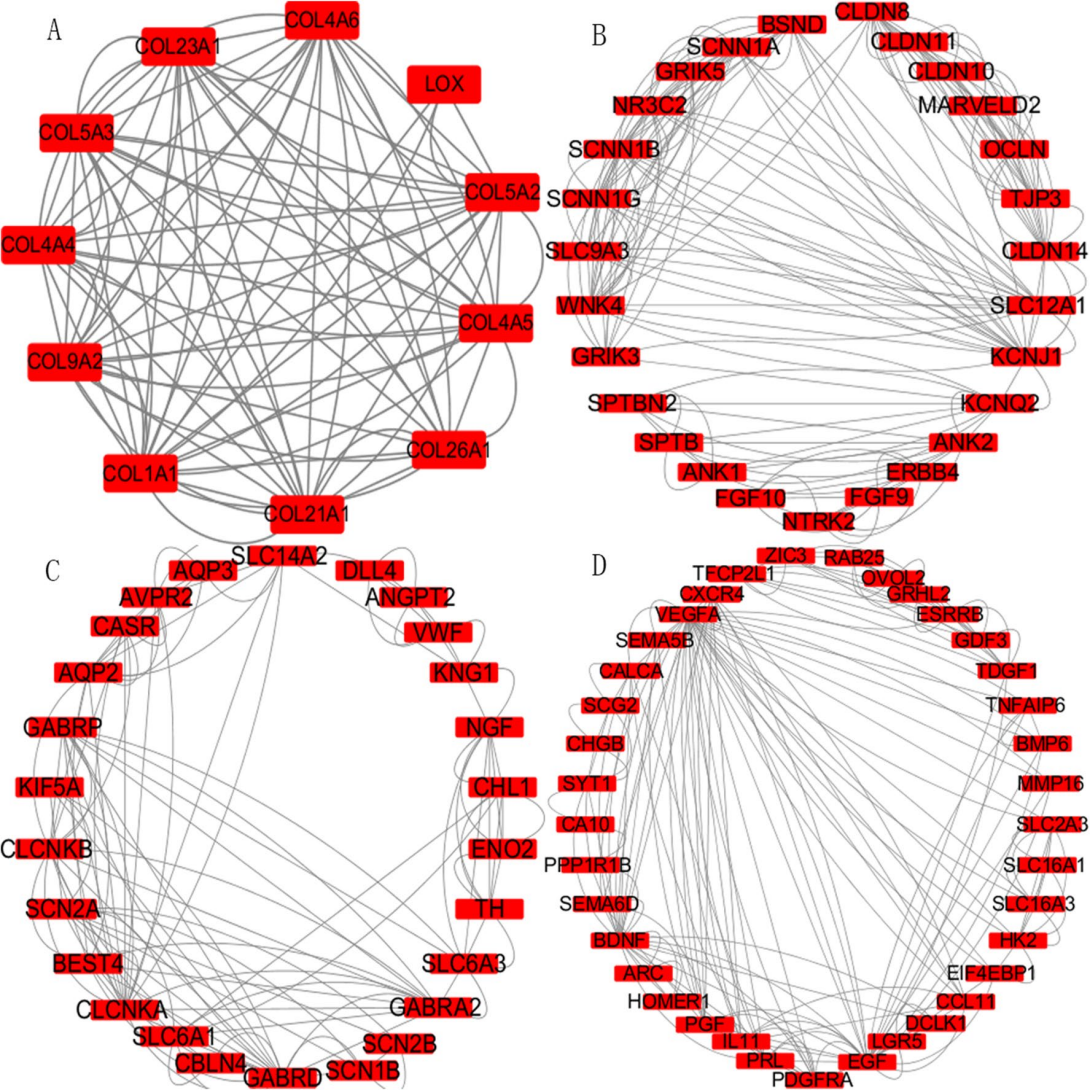
The PPI network is visualized in cytoscape and the MCODE algorithm will modularize the network. The figure shows the four modules filtered by a score greater than 4.5. (**A**) Module 1 11 nodes 88 edges (**B**) Module 2 27 nodes 168 edges (**C**) Module 3 26 nodes 116 edges (**D**) Module 4 37 nodes 164 edges.

A total of 1307 genes with the most significant correlation were obtained by the WGCNA module, imported into the string database, and visualized using cytoscape. The MCODE plugin received a total of 20 modules. The four modules with more significant scores were filtered by the filter condition of a score greater than 4.5 (Fig. 3). They are Module 1 (Fig. 3A, 11 nodes with 88 edges), Module 2 (Fig. 3B, 27 nodes with 168 edges), Module 3 (Fig. 3C, 26 nodes with 116 edges), and Module 4 (Fig. 3D, 37 nodes with 164 edges). The nodes in the module are used as key genes, and then a total of 101 key genes are obtained.

### Univariate Cox regression analysis for key prognostic factors

**Figure 4:**
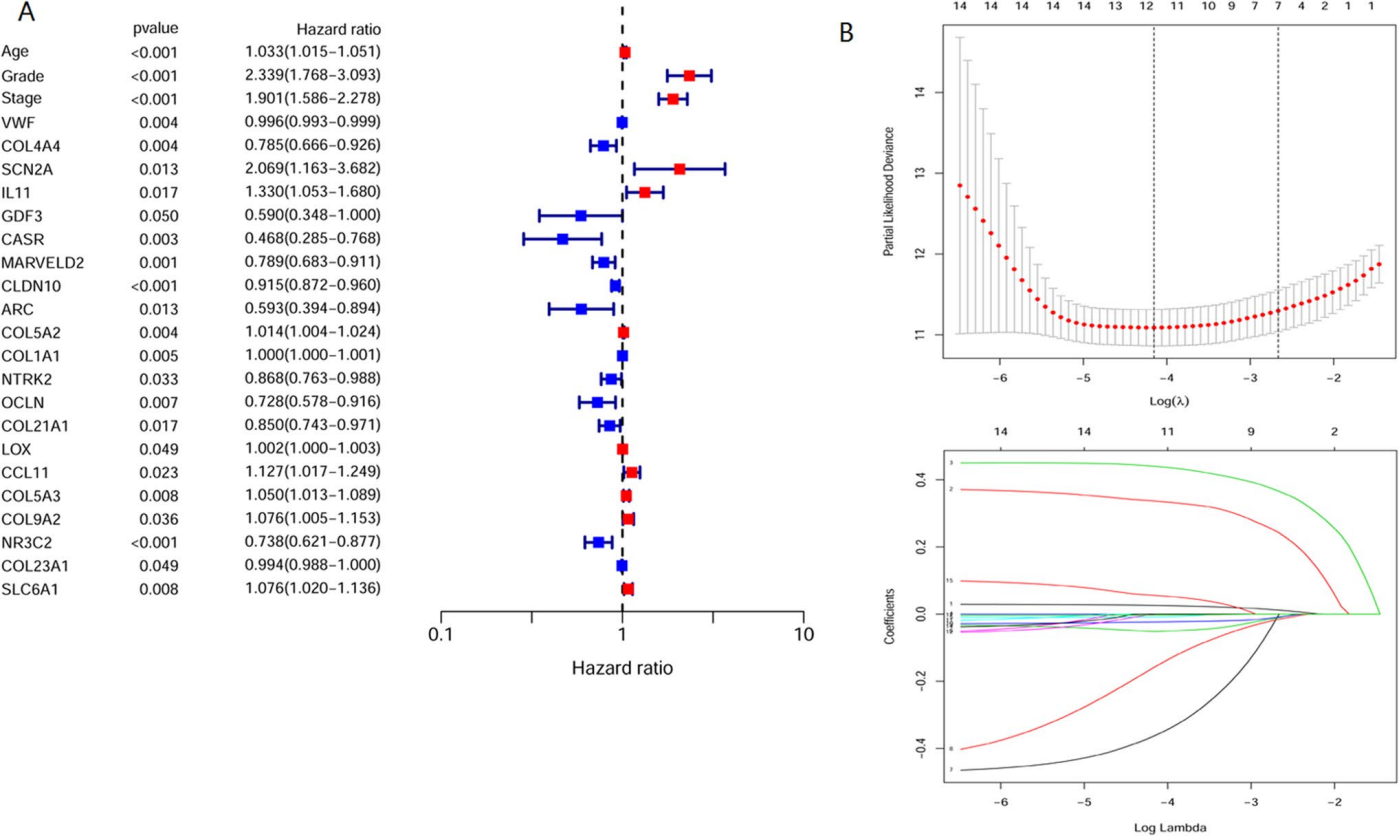
Screening prognostic factors and model construction. (**A**) Univariate Cox regression was used to screen 24 factors associated with KIRC prognosis, including three clinical features and 21 key genes. Blue in the figure indicates a negative correlation, and red indicates a positive correlation. (**B**) Parameter selection and coefficient determination for model construction. Seven key factors were filtered by lambda. Model construction for the LASSO regression included: age, grade, stage, GDF3, CASR, CLDN10, and COL9A2. The coefficient distribution plot calculates a coefficient for each gene, indicating each factor’s weight in the model.

Twenty-four prognostic key factors, including three clinical traits (age, grade, stage) and 21 genes, were screened by univariate Cox regression analysis (Fig. 4A). Eleven of these factors are risk factors, and 13 are protective factors. GEPAI further validated the 21 genes, and seven genes (SCN2A, COL5A2, COL1A1, NTRK2, LOX, COL5A3, SLC6A1) that were not significantly associated with survival (*p* > 0.05) were deleted. The remaining 14 prognostic key genes and three clinical traits were used for model construction.

### Construction of the model

Lasso regression was performed on the key prognostic factors, and seven factors were screened for model construction (Fig. 4B), including age, grade, stage, GDF3, CASR, CLDN10, and COL9A2. The first three are clinical characteristics, and the last four are key genes. Based on the coef value of each key factor, the risk score can be calculated for each sample. The formula is: risk core = age * 0.0313014315935757 + grade * 0.389724551232819 + stage * 0.457790507453084 + GDF3 * − 0.51027183151356 + CASR * − 0.483914624102906 + CLDN10 * − 0.0382983311463935 + COL9A2 * 0.081731554956538. The sample was divided into high-risk and low-risk groups based on all samples’ median value of risk scores.

**Figure 5:**
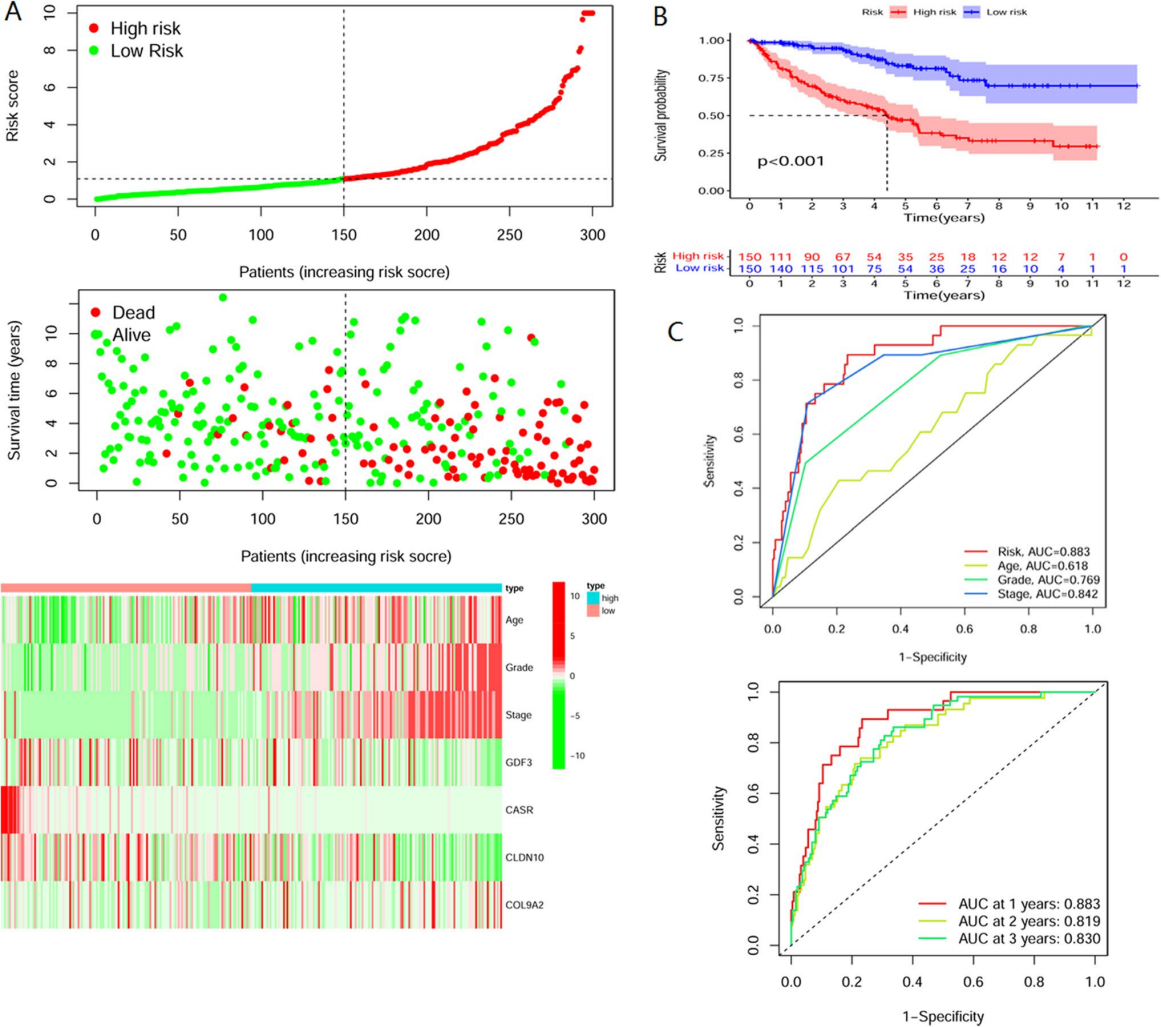
Evaluation of model effects. (**A**) The risk curves are ordered by patient risk, with the left side of the dotted line indicating the low-risk group and the right side indicating the high-risk group. Green points indicate patient survival, and red points indicate patient death. (**B**) Patients were divided into high-risk and low-risk groups, with blue, indicating the low-risk group and red indicating the high-risk group. The survival curves of the two groups are significantly different. (**C**) ROC curves. The area under the curve indicates the model’s accuracy in predicting survival status. The red curve Risk in the figure indicates the accuracy of model prediction in this study, which is higher than the prediction accuracy of age, grade, and stage.

Risk curves were plotted for the sample in descending order of risk score, and it was found that the high-risk group had significantly more deaths than the low-risk group. The heat map illustrates that the expression of key factors involved in the construction of the model differed considerably in the high and low-risk groups (Fig. 5A). After dividing the sample into high- and low-risk groups, the survival analysis showed a worse prognosis in the high-risk group (Fig. 5B). They were statistically significant (*p* < 0.001). The Cox curve also showed that the model’s accuracy in predicting patient survival status at 1, 2, and 3 years was 0.883, 0.819, and 0.830, respectively, and was higher than other clinical traits (Fig. 5C).

After the inclusion of clinical traits to construct the model, it was found that the model’s accuracy was significantly improved compared with that before the inclusion. The models were compared with those made by other researchers in KIRC, as shown in Table 1. Most of the datasets used by the researchers to construct the models were TCGA datasets, but the gene sets used to construct the models were all different, and the area under the ROC curve varied, with the highest being 0.788. In this study, the model was constructed by adding clinical traits to the key genes, and the model’s prediction accuracy was improved substantially, reaching 0.883.

**Table 1:** Comparison of models.

	Model	DATA set	AUC 1-year
Our model	Age + grade + stage + GDF3 + CASR + CLDN10 + COL9A2	TCGA	0.883
Liu et al. 05	ALDH6A1 + FBP1 + HAO2 + TYMP + PSAT1 + IL4I1 + P4HA3 + HK3 + CPT1B + CYP26A1	TCGA	0.708
Meng 09	C4orf19-69,001-AT + C16orf13-32,924-ES + KIAA0930-62645-AP + FAM120C-89237-AT + UACA-31439-AP	TCGA	0.788
Huang12	ALDOB + ESRRG + EFHD1	TCGA	0.717
Hu 13	BID + CCNF + DLX4 + FAM72D + PYCR1 + RUNX1 + TRIP13	TCGA	0.798
Wu 14	TRIM16 + TRIM32 + TRIM24 + TRIM8 + TRIM27 + PML + TRIM11	TCGA	0.685(5-years)

The expression of genes is different in different stages and grades of the disease, and the means and outcomes of treatment are different. In this study, the models were constructed separately using Lasso regression according to different stages and grades. Stage I, the genetic and clinical characteristics of the constructed models were Age, SCN2A, GDF3, and CASR, with an accuracy of up to 0.952. The stage III model consisted of age, grade, MARVELD2, COL23A1, and SLC6A1 with an accuracy of 0.928. In the grading, the model of G2 was constructed by age, COL4A4, SCN2A, CASR, ARC, NTRK2, CCL11, and SLC6A1 with an accuracy of 0.903. The accuracy of the G3 stage model was 0.864, and the factors included stage, COL9A2, and NR3C2. The analysis revealed that multiple models used the three factors age, SCN2A, and CASR, indicating that they are essential features in the onset and progression of the disease. Also, the models constructed by stage and grade are more accurate, indicating a better disease prediction (Table 2).

**Table 2:** Model construction for different grades and stages.

	Model	Model Parameters	AUC 1-year
Stage I	Age + SCN2A + GDF3 + CASR	0.0463313068688194 * age + 1.48114093662391 * SCN2A − 1.2305972303024 * GDF3 − 1.18297850827046 * CASR	0.952
Stage III	Age + grade + MARVELD2 + COL23A1 + SLC6A1	0.0514169815936983 * age + 0.799106874828387 * grade − 0.278733316708886 * MARVELD2 − 0.0189914177693838 * COL23A1 + 0.161249574331587 * SLC6A1	0.928
G2	Age + COL4A4 + SCN2A + CASR + ARC + NTRK2 + CCL11 + SLC6A1	0.100569124886711 * age − 0.4289258084134 * COL4A4 + 3.51304788253637 * SCN2A − 0.757315594407417 * CASR − 1.41094376096415 * ARC − 0.196524945140031 * NTRK2 − 1.24072596854335 * CCL11 + 0.441868367348458 * SLC6A1	0.903
G3	Stage + COL9A2 + NR3C2	0.753703627694245 * stage + 0.14638925018074 * COL9A2 − 0.251774594464014 * NR3C2	0.864

### Test set to verify model validity

**Figure 6:**
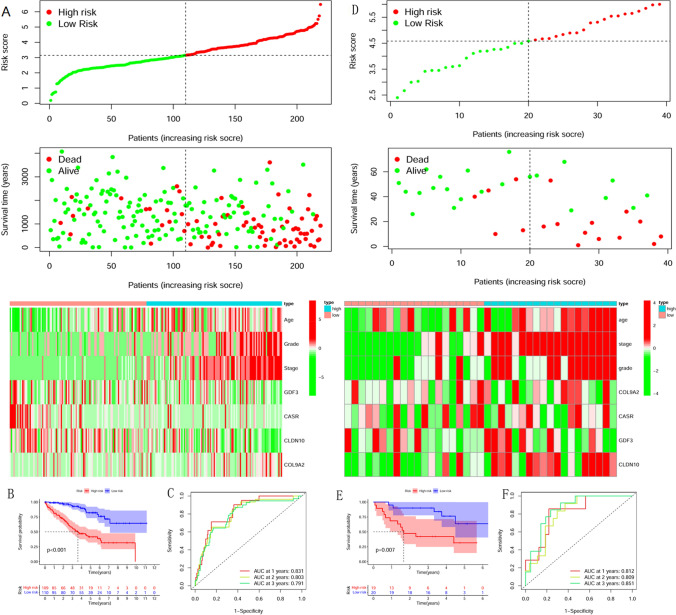
Test Set Validation in R 3.6.2(https://www.r-project.org/). (**A**) TCGA Test Set Risk Curve and Risk Heat Map. (**B**) TCGA Test Set Survival Curve. There was a significant difference in survival between the high and low-risk groups. (**C**) TCGA Test Set ROC Curve. The prediction accuracy for survival at 1, 2, and 3-years was 0.831, 0.801, and 0.791, respectively. (**D**) GSE29609 Test Set Risk Curve and Risk Heat Map. (**E**) GSE29609 Test Set Survival Curve. (**F**) GSE29609 Test Set ROC Curve. The prediction accuracy for survival at 1, 2, and 3-years was 0.812, 0.809, and 0.851, respectively.

In the TCGA test set, the risk score of each sample was calculated based on the risk score formula, and the sample was divided into high and low-risk groups based on the median value. Survival analysis, risk curves, and risk heat maps were done separately for the high and low-risk groups. There were significant differences in survival time and survival status between the two groups (Fig. 6A). Patients in the high-risk group had a worse prognosis than those in the low-risk group, as shown in Fig. 6B. The ROC curves illustrated that the model also performed well in predicting patient survival status, with areas under the curves of 0.831, 0.801, and 0.791, respectively (Fig. 6C). Better results were also obtained in the GSE29609 test set, with a significant survival difference between the high- and low-risk groups (*p* = 0.007) and higher prediction accuracy of 0.812, 0.809, and 0.851, respectively (Fig. 6D–F). The results indicate that the model is universally adaptable and can be used as a helpful tool to distinguish between high and low-risk groups in other data sets.

**Figure 7:**
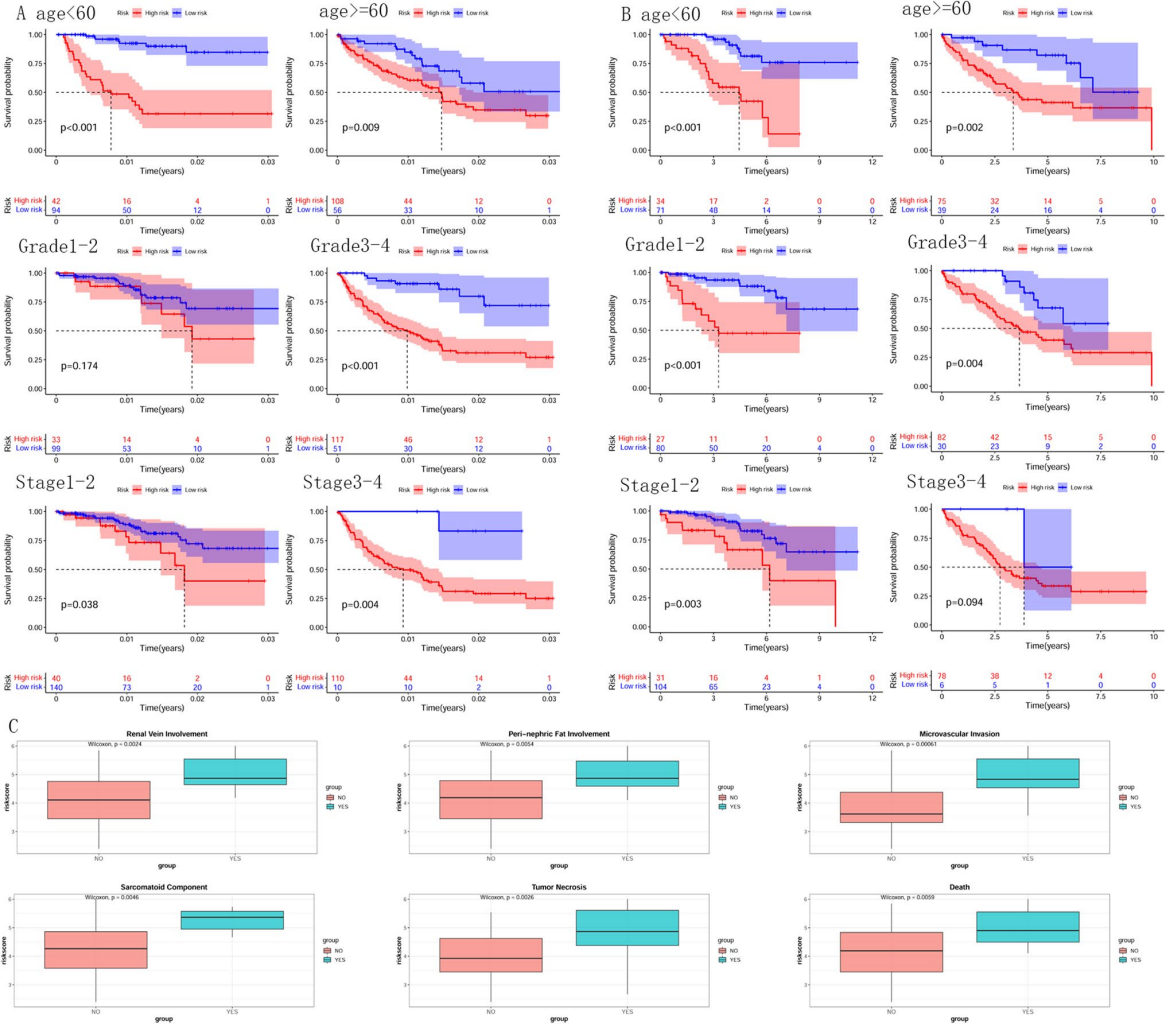
Effectiveness of the model at different ages, grades, and stages. Survival analysis of models in age less than 60-years, age greater than or equal to 60-years, grade1-2, grade3-4, stage1-2, stage3-4. Red indicates the high-risk group, and blue indicates the low-risk group. (**A**) TCGA training set group validation. (**B**) TCGA test set group validation (**C**) GSE29609 test set group validation. Risk scoring was significantly different in subgroups such as renal vein involvement, peri-nephric fat involvement, and microvascular invasion.

The training and test sets of the TCGA dataset were grouped to validate the model further. The data were grouped according to age (> 60, <  = 60), grading (1–2, 3–4), and staging (1–2, 3–4) to do survival analysis. The results showed a significant difference in survival status between the high and low-risk groups in the TCGA training set, except for the subgroup of grades 1–2, where there was no significant difference (Fig. 7A). The same was true in the TCGA test set, where the survival status of the high-risk group was significantly lower than that of the low-risk group (Fig. 7B). It indicates that the model also has a better effect in different subgroups.

In the renal vein involvement, peri-nephric fat involvement, microvascular invasion, sarcomatoid component, tumor necrosis, and death subgroups of the GSE29609 dataset, there were significant differences in risk scoring, with significantly higher scores in progression than in non-progression (Fig. 7C), further indicating that higher risk scores are less favorable for disease progression.

### GSEA enrichment analysis for high and low risk groups

**Figure 8:**
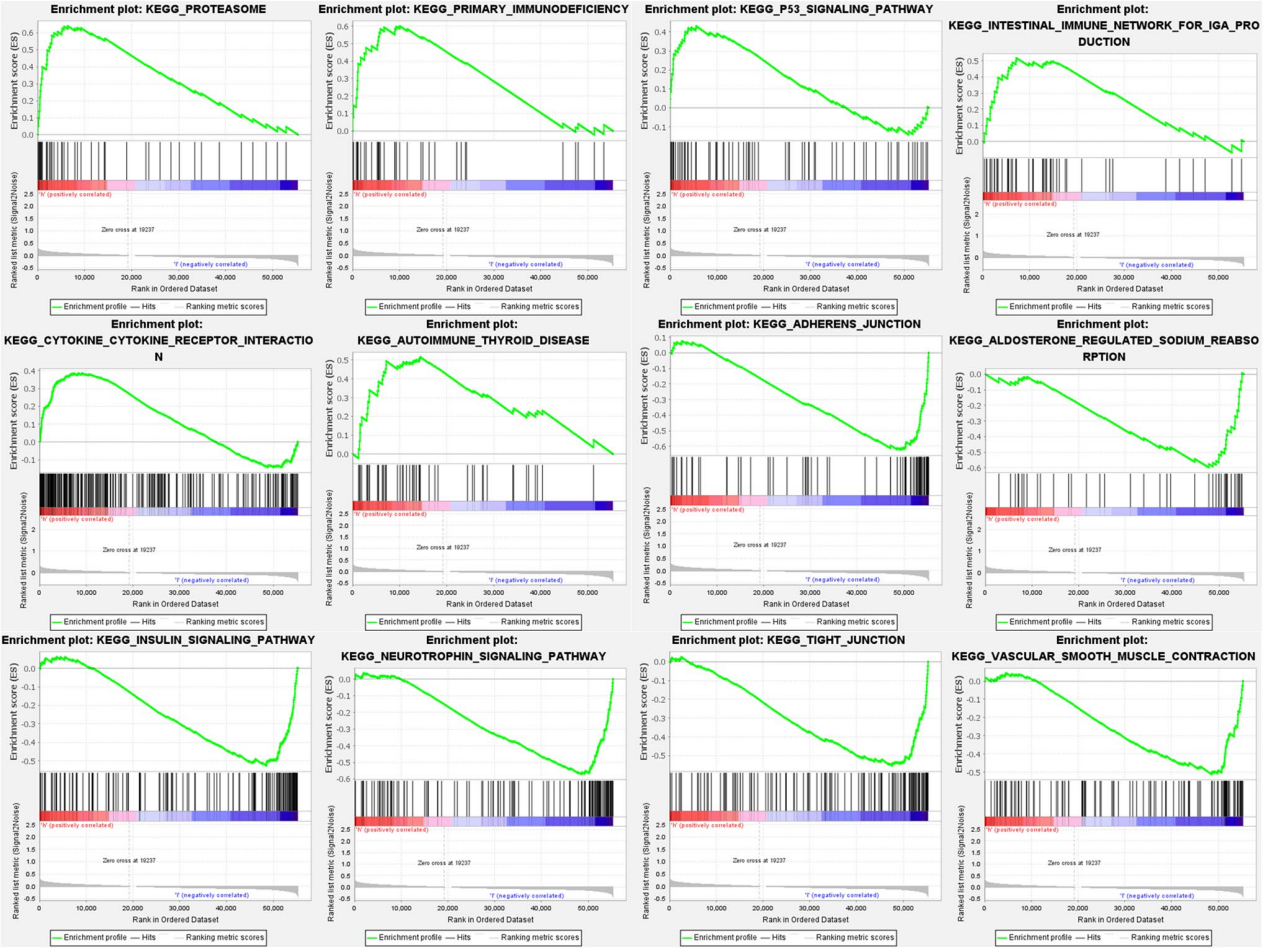
Enrichment analysis of GSEA for high and low-risk groups. The samples were divided into a high-risk group and a low-risk group by model calculations. The differences in the enriched pathways between the two groups were compared by GSEA enrichment analysis. Red indicates the high-risk group, and blue indicates the low-risk group.

GSEA enrichment analysis was done on samples labeled in the high and low-risk groups to see the enriched pathways in both groups. The results showed that the high-risk group was enriched in the proteasome, primary immunodeficiency, cytokine-receptor interactions, the intestinal immune network for IgA production, autoimmune thyroid disease, and the p53 signaling pathway. The low-risk group was mainly enriched in tight junction, neurotrophin, insulin signaling pathway, vascular smooth muscle contraction, adherent junction, and aldosterone-regulated sodium reabsorption (Fig. 8).

Proteasomes are used to regulate specific protein concentrations and to degrade misfolded proteins. It is also involved in the control of cell cycle and transcriptional regulators. Many studies have shown that proteasomes are associated with cancer and that cancer cells have higher levels of proteasomes. The signaling pathway of p53 functions to maintain genomic integrity and regulate cell cycle arrest and apoptosis. Additional studies have shown that p53 regulates autophagic activity, alters metabolism, and inhibits pluripotency and cell plasticity. P53 gene is the gene with the highest relevance to human tumors, with mutations in this gene occurring in more than 50% of malignancies. In tumors, p53 is directly inactivated by mutation or lost through abnormalities in regulatory pathways.

### Immunological analysis of high and low risk groups

**Figure 9:**
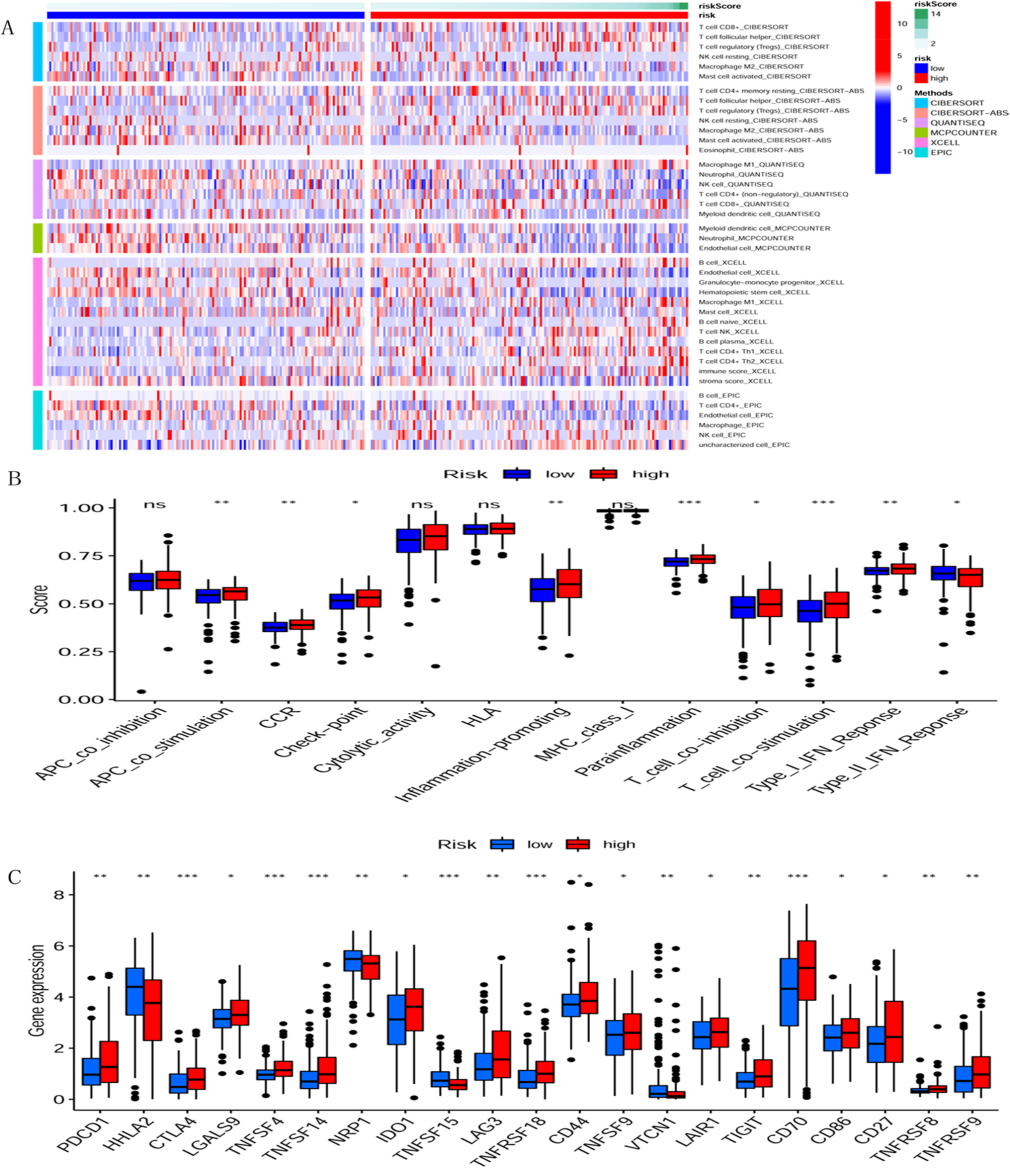
Immunological analyses. Model calculations divided the samples into high-risk and low-risk groups. The two groups were analyzed for differences in Immune cell infiltration, Immune function analysis, and Immune checkpoint. (**A**) Immune cell infiltration analysis. Calculate the correlation between immune cells and the two groups of samples at high and low risk by six different algorithms. (**B**) Immune function analysis. The differences between high and low-risk groups in different immune functions were analyzed. Where blue indicates the low-risk group and red indicates the high-risk group. (**C**) Immune checkpoint analysis. The differences in the expression of immune checkpoints in the high and low-risk groups were analyzed, where blue indicates the low-risk group and red indicates the high-risk group.

The immune cell infiltration results file and the sample grouping file were combined to calculate the correlation between immune cells and the two groups of high and low risk of the sample. The analysis results are shown in Fig. 9A, and the immune cell correlation heat map demonstrates the results obtained from six different calculations. The results of the heat map showed that CD8(+) T cell, T follicular helper cell, regulatory T cell, B cell, and M1 macrophage were upregulated in the high-risk group, while natural killer (NK) cell, CD4(+) memory T cell, mast cell activated, myeloid dendritic, endothelial cell was upregulated in the low-risk group. Also, the immune score was elevated in the high-risk group, while the opposite was true for the stroma score.

Differential immune function analysis showed APC co-stimulation, CC chemokine receptor(CCR), check-point, inflammation-promoting, part inflammation, T cell co-inhibition, T cell co-stimulation, and type I IFN response were significantly differentially expressed in high and low-risk groups and scored higher and more active in the high-risk group. Moreover, type II IFN response scored higher in the low-risk group than in the high-risk group (Fig. 9B).

Immune checkpoint differential analysis showed PDCD1, CTLA4, LGALS9, TNFSF4, TNFSF9, TNFSF14, IDO1, LAG3, CD44, LAIR1, TIGIT, CD70, CD86, TNFRSF8, TNFRSF9, TNFRSF18 were upregulated in the high-risk group. In contrast, HHLA2 NRP1 TNFSF15 VTCN1 showed the opposite behavior in the high and low expression groups (Fig. 9C).

## Conclusion

In this study, the prognostic model of KIRC was obtained by bioinformatics analysis. This study consists of three main parts: model construction, validation, and analysis. In this study, clinical features were added to the construction of the model, thus improving its accuracy. The model’s accuracy in predicting the 1-year, 2-year, and 3-year survival rates of KIRC patients was 0.883, 0.819, and 0.830, respectively, which has higher accuracy and better stability than the prediction model constructed by genes only. It can help physicians treat patients more precisely, resulting in a better prognosis. Our study found that GDF3, CASR, CLDN10, and COL9A2 are key genes for KIRC prognosis and essential for model construction. In the model validation part, we used two datasets, the TCGA test set and the GSE29609 test set. In both datasets, the model predicted better. In the TCGA test set, the accuracy of predicting 1-, 2-, and 3-year survival of KIRC patients was 0.831, 0.801, and 0.791, respectively. 0.812, 0.809, and 0.851 in the GSE29609 test set, respectively. The better performance in different datasets indicates that the model is scalable. In more KIRC clinical datasets, the model can be used as a reliable means for accurate prediction and evaluation of patients.

Although this study improved the accuracy of the KIRC prognostic model, it is still far from accurate prediction, and the accuracy can be further enhanced. In constructing the KIRC predictive model, we used only a small amount of clinical information, such as gender, age, stage, and grade, due to the limitation of the completeness of the TCGA dataset. The clinical data related to patient prognosis (age, stage, and grade) was screened out by univariate COX regression analysis as elements of the model construction. The model would have worked better if more clinical information had been used in its construction. For example, submitting information such as body temperature, heart rate, respiration, mean arterial pressure, electrolytes, total white blood cell count, and chronic disease count into the model construction can provide more information to the model, which is very beneficial to the model construction. Meanwhile, clinical information such as metastasis and recurrence is also critical, which directly suggests the progress of the disease course and will play a very positive role in improving the model’s accuracy.

Additional helpful information can be added to the construction of the KIRC prognostic model, which may also improve the accuracy and usefulness of the model. For example, patient imaging data can be added to the model construction, including CT, ultrasound, MRI, and other imaging data. First, image features (shape, size, texture, density) are extracted from the image data. The patient’s prognosis is combined to obtain the prognosis-related imaging features. Finally, the patient’s imaging features and genetic and clinical information are combined to construct a prognostic model jointly. Since the model combines information from multiple aspects of the patient, the model thus obtained will be more objective, accurate, and practical.

Although a better prognostic model construction of KIRC was obtained in this study, many areas could be improved. In conclusion, this will help the study of KIRC and provide some guidance for the diagnosis, treatment, and prognosis of KIRC.

## Data Availability

The data used in this study are public. The dataset downloaded from the GEO database is GSE29609. TCGA Database: https://portal.gdc.cancer.gov/. GEO Database: https://www.ncbi.nlm.nih.gov/geo/. GSE29609: https://www.ncbi.nlm.nih.gov/geo/query/acc.cgi?acc=GSE29609.
